# Cyanobacterial Cyclic Peptides Can Disrupt Cytoskeleton Organization in Human Astrocytes—A Contribution to the Understanding of the Systemic Toxicity of Cyanotoxins

**DOI:** 10.3390/toxins16090374

**Published:** 2024-08-23

**Authors:** Anja Bubik, Robert Frangež, Monika C. Žužek, Ion Gutiérrez-Aguirre, Tamara T. Lah, Bojan Sedmak

**Affiliations:** 1 Department of Genetic Toxicology and Cancer Biology, National Institute of Biology, Večna pot 121, SI-1000 Ljubljana, Slovenia; tamara.lah@nib.si (T.T.L.); bojan.sedmak@fvo.si (B.S.); 2 Faculty of Environmental Protection, Trg mladosti 7, SI-3320 Velenje, Slovenia; 3 Institute of Preclinical Sciences, Veterinary Faculty, University of Ljubljana, Gerbičeva 60, SI-1000 Ljubljana, Slovenia; robert.frangez@vf.uni-lj.si (R.F.); monika.zuzek@vf.uni-lj.si (M.C.Ž.); 4 Department of Biotechnology and Systems Biology, National Institute of Biology, Večna pot 121, SI-1000 Ljubljana, Slovenia

**Keywords:** astrocytes, cyclic cyanobacterial peptides, cytoskeletal organization, Ser/Thr phosphatases, systemic toxicity

## Abstract

The systemic toxicity of cyclic peptides produced by cyanobacteria (CCPs) is not yet completely understood. Apart from the most known damages to the liver and kidneys, symptoms of their neurotoxicity have also been reported. Hepatotoxic CCPs, like microcystins, as well as non-hepatotoxic anabaenopeptins and planktopeptins, all exhibit cytotoxic and cytostatic effects on mammalian cells. However, responses of different cell types to CCPs depend on their specific modes of interaction with cell membranes. This study demonstrates that non-hepatotoxic planktopeptin BL1125 and anabaenopeptins B and F, at concentrations up to 10 µM, affect normal and tumor human astrocytes (NHA and U87-GM) in vitro by their almost immediate insertion into the lipid monolayer. Like microcystin-LR (up to 1 µM), they inhibit Ser/Thr phosphatases and reorganize cytoskeletal elements, with modest effects on their gene expression. Based on the observed effects on intermediate filaments and intermediate filament linkage elements, their direct or indirect influence on tubulin cytoskeletons via post-translational modifications, we conclude that the basic mechanism of CCP toxicities is the induction of inter- and intracellular communication failure. The assessed inhibitory activity on Ser/Thr phosphatases is also crucial since the signal transduction cascades are modulated by phosphorylation/dephosphorylation processes.

## 1. Introduction

Toxic cyanobacteria are increasingly being perceived as a potential health hazard since exposure to cyanobacterial toxins can cause serious environmental and human health effects. The incidences of cyanobacterial blooms have been increasing worldwide, and with warming climates and constant intake of nutrients from human activities, this problem would continue to increase and threaten drinking and recreational water sources even more (e.g., [1,2,3,4]).

Microcystins (MCs) are the most widely distributed and toxic cyanobacterial toxins, posing a serious threat to human health, and have also been recognized by the World Health Organization (WHO) as an emerging public health issue [5]. The tragic hepatotoxic poisoning by MCs that occurred in Caruaru, Brazil, in 1996 opened several questions about the possible toxic effects of these biologically active substances that are produced by cyanobacteria. Fifty-two of sixty victims of this intoxication were confirmed to have been intravenously exposed to lethal concentrations of hepatotoxic cyclic cyanobacterial peptides (hCCPs), which include MCs. Apart from the damage to the liver, all these patients developed acute signs of neurotoxicity [6,7]. These victims were exposed to a mixture of biologically active cyanobacterial products and especially MCs. It is already known that some other cyanobacterial peptides can also be produced in significant amounts in bloom-forming cyanobacteria [8]. Additionally, cyanobacterial extracts that contain MCs as their main components are significantly more biologically active compared to the same concentrations of isolated MCs themselves. Indeed, Ding et al. [9] suggested that other biologically active contaminants in microcystic extracts might promote the microcystin activities, potentially through assisted cell entry, to thus provide synergistic effects. The high lethality of hCCPs, such as MCs, is due to two major characteristics: they are potent and irreversible inhibitors of Ser/Thr protein phosphatase activity, e.g., [10], leading to increased phosphorylation of proteins in liver cells, which can impact metabolic pathways or cell division [11]. They show organotropism as a result of their misuse of the active transport systems of particular cells and organs, e.g., [12].

The precondition for toxicity of biologically active substances is for them to come into intimate contact with the basic physiological processes of the organism. At the cellular level, this means that biologically active substances must either interact with the cell surface and its various receptors or must pass through the cell membrane into the interior of the cell. The liver and kidneys are organs where membrane-transport mechanisms have crucial roles in detoxification and elimination of xenobiotics. The organotropism and eventual lethality of hCCPs are therefore possible due to their unimpeded, or even active, entry into specific organs that are composed of determined cell types, which can lead to uncontrolled hyperphosphorylation of a variety of cell proteins, e.g., [13]. Due to their specific molecular structure, for cell entry, hCCPs require specific organic anion transporting polypeptides/solute carrier organic anion transporters (OATPs/SLCOs) [12,14]. However, such transport systems have not been detected in numerous cell types, including established cell lines and immortalized cells, such as immortalized hepatocytes, e.g., [15,16]. Despite this, cells that cannot actively take up hepatotoxic MCs can still undergo morphological and physiological changes when exposed to higher MC concentrations than those used in studies on primary hepatocytes [17]. Therefore, there is the need to strictly distinguish between the lethal effects of hCCPs that result from their active entry into cells via transporters and their cytotoxicity due to interactions with the membrane and/or receptors of the cells.

In addition to the well-known and most extensively studied hCCPs (e.g., MCs) non-hCCPs of similar molecular mass and structure are frequently produced in significant amounts by bloom-forming cyanobacteria [8,18]. These include the 3-amino-6-hydroxy-2-piperidone-containing depsipeptides, such as the planktopeptin BL1125 (PP BL1125) [19] and anabaenopeptins B and F (AnP-B, AnP-F), as well as cyclic peptides with an ureido linkage [20]. Furthermore, several non-hCCPs (e.g., AnPs and anabaenopeptilides) also show protein phosphatase inhibition [21], and some (e.g., AnPs and PP BL1125) are also cytotoxic in addition to their other biological activities [22]. Characteristic of various AnPs is the inhibition of carboxypeptidases [23], including carboxypeptidase TAFIa [24], while depsipeptides are particularly known as serine protease inhibitors [25].

Therefore, the aim of this study was to evaluate the effects of three non-hepatotoxic cyclic cyanopeptides (PP BL1125, AnP-B, and -F) and hepatotoxic MC-LR on human astrocytes in vitro, using cellular assays and molecular analysis, to better understand their role and contribution to the systemic toxicity of cyanotoxins. Our attention was focused on interactions of selected non-hCCPs at the level of the cell membrane and the consequent reorganization of specific intermediate filaments, which in the case of MCs are the major indirect target of the Ser/Thr protein phosphatase inhibition [26], their influence of on tubulin post-translational modifications [27], and the tubulin antiproliferative activity [22] that could contribute to systemic intoxication.

## 2. Results

### 2.1. Interactions of CCPs with the Lipid Monolayer

**Figure 1:**
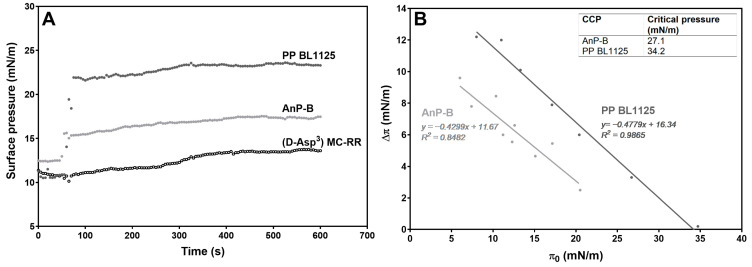
Interactions of CCPs with the lipid monolayer. (**A**) Binding kinetics of PP BL1125, AnP-B, and [D-Asp^3^]-MC-RR (final concentrations of 20 µM) to dioleylposphatidylcholine: cholesterol (70:30) monolayers, measured at the initial surface pressure (Π_i_) of ~11 mN/m; (**B**) extrapolation of zero pressure variation (ΔΠ = 0) for the critical pressure of insertion of the CCPs tested.

Interactions with an air–water interface and insertion into a lipid monolayer are techniques that are widely used to characterize interactions with lipid membranes of toxic (or otherwise) biologically active proteins and peptides [28,29]. The depsipeptide PP BL1125 progressively interacted with the air–water interface up to a concentration of 25 μM, where saturation was observed. This indicated an amphipathic character of non-hCCPs, as expected from their molecular structures [19]. The insertion kinetics of PP BL1125, AnP-B, and [D-Asp^3^]-MC-RR into the lipid monolayers of dioleylposphatidylcholine/cholesterol (70:30) at an initial pressure of ~11 mN/m are shown in Figure 1A. [D-Asp^3^]-MC-RR showed little membrane insertion. However, some degree of interaction can be assumed on the basis of the slow but persistent increase in surface pressure during this experiment. Both non-hCCPs tested (i.e., PP BL1125 and AnP-B) interacted rapidly with the monolayer, where a higher final increase in the surface pressure was seen for PP BL1125 compared to AnP-B (Figure 1A).

A lateral pressure of 30 mN/m is typically assigned to the lipids that form part of natural lipid bilayers. The critical pressures of insertion for the two non-hCCPs were determined by extrapolation to the initial zero pressure variation (i.e., ΔΠ = 0) (Figure 1B). This provided critical pressures of 34.2 mN/m for PP BL1125 and 27.1 mN/m for AnP-B, which lie close to (AnP-B) and beyond (PP BL1125) the assigned 30 mN/m. This indicates their potential for insertion into natural membranes and particularly for PP BL1125.

### 2.2. Protein Phosphatase Inhibition

The PP1 and PP2A activities were inhibited by all the CCPs tested. The non-hCCPs PP BL1125, AnP-B, and AnP-F showed weaker inhibitory activities compared to the hepatotoxic MC-LR. Hydrolysis of the *p*-nitrophenyl phosphate substrate by PP1 and PP2A was potently inhibited by MC-LR, with IC_50_ values in the nanomolar range, whereas PP BL1125, AnP-B, and AnP-F inhibited both Ser/Thr protein phosphatases in the micromolar range (Table 1).

### 2.3. Metabolic Activity

The metabolic activity of the NHAs was significantly decreased after exposure to MC-LR for 24 h. At the highest concentration of 10 µM MC-LR, the cell viability was ~20% lower than the control, whereas all three of the non-hCCPs tested here (i.e., PP BL1125, AnP-B, and AnP-F) showed no significant changes from the control (Figure S1A).

The viability of the U87-MG cells (cell line derived from human glioma) exposed to up to 10 µM MC-LR or the non-hCCPs was not significantly changed; although a mean 15% decrease in viability was seen for 10 µM MC-LR, this did not reach statistical significance. Furthermore, the low concentrations of all three non-hCCPs showed opposite effects on the U87-MG cell line, where there was an increase in metabolic activity (Figure S1B). For further characterization of the gene expression response, only concentrations of CCPs that resulted in minimal cytotoxicity were used.

### 2.4. Gene Expression Analysis

We observed different *ACTB* and *TUBA1A* expression profiles between the two cell lines used. *TUBA1A* relative gene expression prevailed in NHAs (Figure S2A), in contrast to the higher *ACTB* relative gene expression in U87-MG cells (Figure S2B). *NES* relative expression was similar in these cell lines, whereas *GFAP* relative expression could not be calculated because it was too low (Figure S2). For both cell lines, gene expression of the OATP/SLCO family members *SLCO1A2*, *SLCO1B1*, and *SLCO1B3* was not detected using the quantitative real-time PCR.

Among all the CCPs tested, only MC-LR did not influence the expression of any gene in NHAs; however, it influenced the expression of *ACTB* and *GFAP* in U87-MG cells. In contrast, non-hCCPs revealed almost no effects on *ACTB* and *GFAP* expressions in both cell lines. The most evident changes were observed for *NES*, where its expression in NHAs was increased after exposure to all three non-hCCPs at all tested doses, while in U87-MG cells, its expression was increased at the highest tested dose of BL1125 and AnP-F. Decreased *TUBA1A* expression was observed at the highest concentration of both anabaenopeptins in NHAs in contrast to U87-MG cells, where all three non-hCCPs statistically significantly increase its expression (Table 2A,B).

### 2.5. Cytoskeleton Organization

The morphology of the NHAs and U87-MG cells were first examined under light microscopy and phase contrast, followed by confocal microscopy.

#### 2.5.1. Light Microscopy

**Figure 2:**
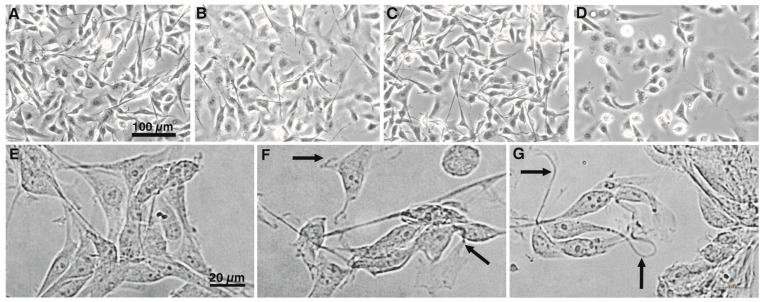
Effects of CCPs on the morphology of the U87-MG cells and NHAs. Representative images of control, non-treated, U87-MG cells (**A**), and U87-MG cells treated with PP BL1125 (**B**), AnP-F (**C**) and [DAsp^3^]-MC-RR (**D**), with each at 20 µM, and of control, non-treated, NHAs (**E**), and cells treated with PP BL 1125 (**F**) and AnP-B (**G**), with each at 1 µM. Arrows, twisted outgrowths from cells (see main text). Magnification (phase contrast): 20× (**A**–**D**); 100× (**E**–**G**).

Light microscopy did not reveal any major morphological changes for the U87-MG cells, even when exposed for 24 h to the highest applied concentration (20 µM) of each of the non-hCCPs (Figure 2B,C). Under the same conditions, exposure to the cytolytic effects of MC-LR resulted not only in loss of astrocytic domain organization, but also in a reduction in cell numbers (Figure 2D). On the other hand, the exposure of the NHAs to PP BL1125 and AnP-B resulted in the formation of twisted astrocytic outgrowths that were clearly noted at higher magnification (100×) and were evident already at 1 µM PP BL1125 and AnP-B (Figure 2F,G).

#### 2.5.2. Cytoskeleton Immunostaining

**Figure 3:**
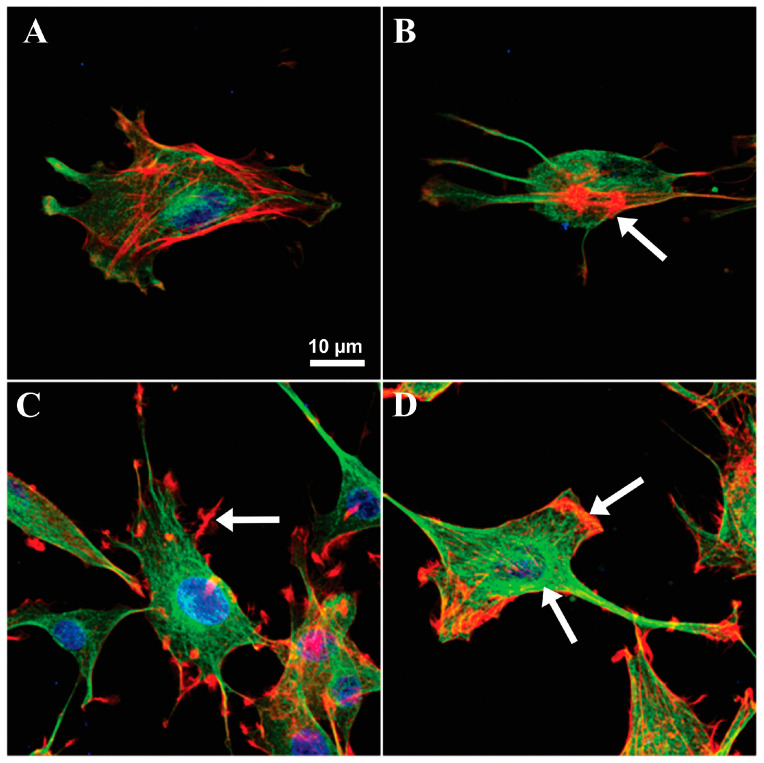
The reorganization of microfilaments and microtubules in NHA and U87-MG cells exposed to the cyclic depsipeptide PP BL1125. Control micrographs of NHA (**A**) and U87-MG (**C**) showing normal distribution of MFs (red) and MTs (green). Reorganization of MFs and MTs is present after 24 h exposure to 10 µM PP BL1125 in NHA (**B**) and U87-MG (**D**). Microtubules were visualized by immunostaining with anti-α-tubulin antibody followed by secondary antibodies labeled with Alexa-488-goat anti-mouse IgG. Actin microfilaments were stained with rhodamine-conjugated phalloidin and cell nuclei with To-Pro-3 (blue). Images were obtained using laser scanning confocal microscopy. Arrows indicate typical cytoskeletal structures in non-treated cells (**C**) and cytoskeletal changes, observed after treatment with PP BL1125 (**B**,**D**).

**Figure 4:**
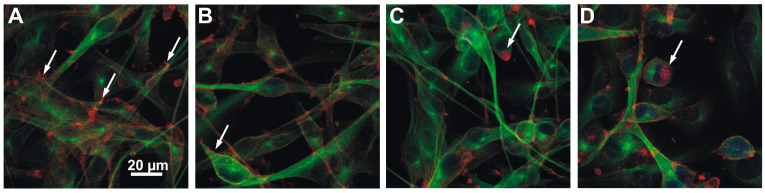
The reorganization of microfilaments and microtubules in cells U87-MG exposed to CCPs. Cells were treated with the PP BL1125 (**B**), AnP-F (**C**), and [DAsp^3^] MC-RR (**D**) in 10 µM concentrations for 24 h, respectively, and compared with control, non-treated cells U87-MG (**A**). Images show the organization of MFs (red) and MTs (green). Microtubules were visualized by immunostaining with anti-α-tubulin antibody followed by secondary antibodies labeled with Alexa-488-goat anti-mouse IgG. Actin microfilaments were stained with rhodamine-conjugated phalloidin and cell nuclei with To-Pro-3 (blue). Images were obtained using laser scanning confocal microscopy. Arrows indicate typical cytoskeletal structures in non-treated cells (**A**) and cytoskeletal changes, observed after treatment with CCPs (**B**–**D**).

Confocal microscopy revealed additional major changes in the microfilament and microtubule organization with exposure of the NHAs and also U87-MG cells to both hCCPs and non-hCCPs. The organization of the microfilaments (MFs) and microtubules (MTs) was markedly changed in the NHAs after treatment with the non-hCCPs, where the morphological changes were much more pronounced compared to those of the U87-MG cells (Figure 2 and Figure 3). In contrast to the U87-MG glioblastoma cells, PP BL1125 generated strong cytolytic effects on the NHAs, with the collapse of the microfilaments in the center of the cell (Figure 3B, red, arrow). In unchallenged U87-MG cells, the microfilaments were organized into specific structures, known as membrane ruffles (Figure 3C and Figure 4A, red, arrows). After 24 h exposure to PP BL1125, there was aggregation of the cortical microfilaments along the basal membrane and degradation of the membrane ruffles (Figure 3D, arrow above; Figure 4B, arrow).

There was also actin aggregation after exposure to AnP-F and [D-Asp^3^]-MC-RR, where the fragmented filaments collapsed into a single perinuclear locus (Figure 4C,D, arrows). The hepatotoxic [D-Asp^3^]-MC-RR resulted in evident reorganization of the MFs (Figure 4D, red) and MTs (Figure 4D, green) in both the NHAs and U87-MG cells. In U98-MG, after 24 h exposure to [D-Asp^3^]-MC-RR, the microfilaments collapsed to the center, around the cell nucleus (Figure 4D, arrow). The microtubules were also reorganized after exposure to the non-hCCPs (Figure 3D and Figure 4B,C, green), but not so clearly as when treated with [D-Asp^3^]-MC-RR (Figure 4D). For the U87-MG cells exposed to PP BL1125, the microtubules were reorganized and concentrated around the nucleus, and aggregated with a star-like appearance beneath the plasma membrane (Figure 4B, arrow).

**Figure 5:**
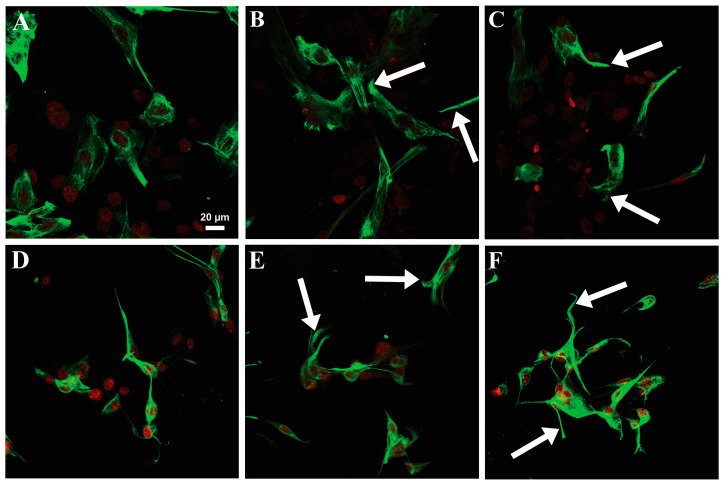
Effects of CCPs on the organization of nestin in NHA (**A**–**C**) and U87-MG (**D**–**F**) cells. Cells were exposed to the cyclic depsipeptide PP BL 1125 (**B**,**E**) and the microcystin MC-LR (**C**,**F**) in 1 µM concentrations for 24 h, respectively, and compared with the control cells (**A**,**D**). Nestin was visualized by immunostaining with anti-nestin antibody followed by secondary antibodies labeled with Alexa-488 goat anti-rabbit IgG (green), and nuclei were marked with To-Pro-3 (red). Images were obtained using laser scanning confocal microscopy. Arrows indicate typical changes observed after treatment with CCPs.

The effects of the CCPs on intermediate filaments were examined with immunolabeling of the two types of intermediate filaments: nestin and GFAP. With exposure to the CCPs, the shapes of the NHAs and U87-MG cells changed. There was aggregation of the cells into clusters, with their outgrowths extended and typically twisted (Figure 2F,G, arrows). Nestin (Figure 5, green) was concentrated in these twisted astrocytic outgrowths and along the cells (Figure 3B,C,E,F, arrows). The most intense nestin concentration was in the NHAs after exposure to MC-LR (Figure 3C). Extremely low concentrations of GFAP were observed in the NHAs, and therefore, the effects of these CCPs on GFAP organization could not be evaluated precisely. However, there were characteristic twisted outgrowths, especially after treatment with AnP-B. For the U87-MG cells, concentration of the GFAP filaments was observed along the entire cell body after exposure to PP BL1125 and MC-LR and in the central part of the cells after exposure to AnP-B.

With the methods used here, desmoplakins were successfully detected in HepG2 desmosomes, as the positive control, while both the NHAs and U87-MG cells used here were desmoplakin negative.

## 3. Discussion

The highly expressed organotropism of hCCPs, which include the MCs and nodularins, and their consequent lethality are primarily due to their active accumulation in specific cell types [14,30]. Although there is evidence that various organs are targets of the deleterious effects of MCs, such as the kidney [31,32,33], lung [34,35], intestine [36,37], heart [38], testis [39], and brain [12,40,41,42,43], hepatotoxicity is the most common effect [44,45,46,47]. The liver is the major organ in the body in terms of blood detoxification and purification, and as such, it is the first and most vulnerable organ. However, effects of MCs on other organs have already been described, such as selective cytotoxicity [48] and genotoxicity [49] toward cells in the brain tissue. Exposure of astrocytes to MC-LR causes hyperplasia, with the increased expression of intermediate filaments leading to spatial learning and memory impairment in rats [50]. An in vitro study has shown that some hydrophobic MCs (i.e., MC-LW and MC-LF) also show selective cytotoxicity in rat primary astrocytes, with the induction of cytoskeletal disruption through intermediate filament degradation [31].

Hepatotoxic CCPs are effective in the reorganization of cytoskeletal elements and especially with primary cell lines, as these have higher levels of the OATPs/SLCOs, which are usually lost in established cell lines. Tenfold to 100-fold higher MC concentrations are needed to produce similar effects on cells where the MCs cannot enter or where the cells are poorly susceptible to the biological activity of the MCs [51]. The most toxic MC, MC-LR, shows only negligible lipid membrane penetration, while the more hydrophobic MC-LF and MC-LW have higher membrane insertion pressures and, therefore, can have pronounced effects on membranes [52]. These two more hydrophobic MCs also have more pronounced effects on certain cells, like the cells derived from human epithelial colorectal adenocarcinoma (Caco-2 cells) compared to MC-LR [53].

In the insertion experiments described here, we tested the [D-Asp^3^]-MC-RR as one of the major and typical microcystin representatives synthesized during metalimnetic blooms of the cyanobacteria *P. rubescens* recurrently present in drinking-water reservoirs [8,54]. The poor interaction of MC-LR with membrane lipid monolayers has already been demonstrated, with the conclusion that the microcystin representatives with a more hydrophilic character need transporters to penetrate the biological membranes and, thus, to influence cell metabolism [55]. We can therefore deduce that CCPs can produce cytotoxic effects at two different levels: on cells with reduced transport activity, or even devoid of specific OATPs, CCPs can act at the level of the cell membrane, including membrane receptors, while when they are internalized through specific transporters, CCPs can directly act at the intracellular level.

The present data indicate very weak interactions of the more hydrophilic hCCPs (e.g., [D-Asp^3^]-MC-RR) with the lipid monolayer, which contrasts with the non-hCCPs, where there was promising potential for cell membrane insertion (Figure 1). Both levels of interaction can cause disturbances to the functional relationships in the homeostasis between normal Ser/Thr protein kinases and their phosphatases, which can result in reorganization of intermediate filaments and, consequently, of other cytoskeletal elements. An important mechanism of the toxic actions of MC-LR is the inhibition of Ser/Thr PP1 and PP2A, e.g., [10,44], which is manifested by reorganization of the cytoskeleton, e.g., [13,56,57], for example, at nanomolar MC-LR concentrations in primary human hepatocytes [17]. Although non-hCCPs inhibited both Ser/Thr protein phosphatases at significantly higher concentrations than the MCs (i.e., µM), they can induce similar effects on cell morphology at the same concentrations as the MCs in cells devoid of the corresponding transporters. Microcystin-LR is one of the most potent inhibitors of both PP1 and PP2A, as it inhibits PP1 with an IC_50_ that is 25,000-fold lower and PP2A with an IC_50_ at least 370,000-fold lower than PP BL1125 (Table 1). Compared to the other two non-hCCPs tested, PP BL1125 is the weakest Ser/Thr protein phosphatase inhibitor, with the strongest elastase and chymotrypsin inhibitory activity [22] and the greatest membrane insertion potential (Figure 1). Despite its weak phosphatase inhibitory activity, PP BL1125 is very effective in the reorganization of cytoskeletal elements. Moreover, the non-hCCPs tested can also promote reorganization of cytoskeletal elements at similar concentrations as the hepatotoxic MCs (i.e., µM) in cells devoid of the corresponding transporters (Figure 3, Figure 4 and Figure 5). The two non-hCCPs used in the present study (i.e., PP BL1125 and AnP-F) also share higher inhibitory activities with the MCs and possibly also higher binding affinities for PP2A than PP1 [58] (Table 1).

The reversible phosphorylation has no doubt an important role, but it only seems to be a minor modification for microtubules. The post-translational modifications (PTMs), such as the de/tyrosination cycle that occur on microtubules, are crucial controllers of their properties and functions. Characteristic of many AnPs, including AnP-A and -F, is the inhibition of carboxypeptidase A (CPA) [23], activated thrombin activatable fibrinolysis inhibitor (TAFIa) [24], and also the elusive tubulin tyrosine carboxypeptidase (TCP). The recent identification of vasohibin/SVBP (small vasohibin binding protein) complexes as TCPs that regulate neural cell differentiation [27] helped us to understand the apparent variations between individual experiments. Tubulin tyrosine carboxypeptidase (TCP) is the enzyme that catalyzes the releases of the C-terminal tyrosine from α-tubulin, converting Tyr-terminated to Glu-terminated tubulin. This enzyme (TCP) is associated with microtubules in living cells. However, its activity strongly depends on three essential preconditions: TCP association with microtubules, the association of vasohibin with SVBP (in the case of neural cells) [27], and the current physiological state of the cells. Namely, the cell cultures at confluence (differentiation process) show very low TCP activity in contrast to cells plated in low concentration (high proliferation rate) with high TCP activity [59]. However, since the activity of TCP is also modulated by phosphorylation/dephosphorylation processes, we should not neglect the inhibitory activity of tested CCPs on Ser/Thr protein phosphatases.

The relatively modest influence of non-hCCPs on gene expression confirms that AnP’s main activity is on tubulin PTM or direct binding to microtubules, as in the case of depsipeptide [60]. For the NHAs, there were significant changes in gene expression for the *NES* intermediate filaments after exposure to the non-hCCPs (Table 2A). These non-hCCPs probably act on cells as stressors and consequently stimulate the defensive reactions of the cells, which results in indistinct increased gene expression for intermediate filaments. The term ‘reactive gliosis’ has commonly been used to describe astrocyte activation under stress and is characterized by elevated expression of the genes for intermediate filaments, such as those for GFAP and vimentin, and for re-expression of nestin [61,62].

Desmoplakins are believed to be the first targets of MC-LR in hepatocytes [26]. Due to the low levels of desmoplakins in both astrocyte cell lines here (i.e., NHAs and U87-MG cells), other intermediate filament binding elements might take their place as the potential targets for the non-hCCPs. Elongated cell protrusions, cell cluster formation, and high levels of nestin in twisted cell outgrowths and beneath the cell membrane (Figure 5) show the influence of non-hCCPs on mechanisms and structures that are related to intracellular organization and intercellular communication (Figure 2, Figure 3, Figure 4 and Figure 5). Moreover, changed polarity of cells and the direction of their motion can occur. As the effects determined here were very similar to those described for hepatocytes [26,63], intermediate filaments would also appear to be the important targets of these CCP activities in astrocytes.

The organization of other cytoskeletal elements, such as microfilaments and microtubules, depends heavily on the phosphorylation levels of intermediate filaments and intermediate-filament-associated proteins, as these can integrate with the cytoskeletal filaments that are involved in the formation of intercellular and intracellular contacts [57,64]. Moreover, the study of Toivola and coworkers highlights that IFs extend their roles beyond mechanical support to include organelle positioning, protein targeting, and signaling pathways, contributing to their cytoprotective and tissue-specific functions [65]. Here, the normal astrocytes (i.e., NHAs) were more sensitive compared to the tumor-derived cells (i.e., U87-MG cells), as was already demonstrated by the generation of cytolytic and cytostatic effects of non-hCCPs. Collapse of the organized cytoskeleton, loss of normal cell shape, and arrested cell division are typical reactions to prolonged (i.e., 48 h and 72 h) exposure of these NHAs to PP BL1125 [22]. These effects were much less pronounced in the U87-MG cells. The CCPs caused aggregation of cortical microfilaments and the breakdown of ruffles, which have important roles in cell motility and communication (Figure 3D and Figure 4B–D). The higher sensitivity of normal cells to cyanopeptides compared to tumor-derived cells may be attributed to several cellular or molecular differences between these cell types. Normal cells typically respond more strongly to xenobiotics due to their intact detoxification systems, efficient stress responses, sensitive cellular machinery, higher expression of drug transporters, and strictly regulated cell cycles, while tumor cells have often altered these mechanisms. In toxin exposure scenarios, the heightened sensitivity of normal cells suggests a higher risk of damage to healthy tissues, which has important implications for public health, safety regulations, and the development of therapeutic agents. However, the increased toxicity of these peptides to normal cells significantly limits their therapeutic potential, as effective cancer treatments require selective targeting of tumor cells with minimal harm to healthy tissues.

As *ACTB* expression remained unchanged after exposure of the U87-MG cells to the non-hCCPs (Table 2B), the modifications seen for the microfilament organization might be a result of changes in the polymerization to depolymerization ratios due to the influence of the non-hCCPs on the homeostasis between normal Ser/Thr protein kinases and their phosphatases. In contrast, the microfilament reorganization after exposure of the U87-MG cells to MC-LR might be a consequence direct inhibition of the Ser/Thr protein phosphatases. It has already been demonstrated that microfilament reorganization is not the result of changed levels of polymerized actin and the ratio of globular to fibrillar actin in hepatocytes when they are treated with MC-LR [66,67,68].

As the result of the CCP inhibitory activities here, increased concentrations of microtubulin in the cell periphery and in the perinuclear area can be noticed (Figure 3B,D and Figure 4B–D). Due to the important role of microtubules in cell division, CCPs also disrupt normal cell growth and development. It appears that these changes in microtubule organization, and consequently to the mitotic spindle, lead to the arrest of cell division in these NHAs. The highly dynamic mitotic spindle is among the most successful targets for anticancer therapies, as such specific drugs can suppress microtubule dynamics and lead to mitotic block and apoptosis [69].

In summary, a large body of evidence has accumulated that indicates an active role of astrocytes in various brain functions, such as cell communication, in the blood–brain barrier, and in the visual and auditory systems in vertebrate [70]. As such, astrocytes are potential targets for systemic intoxication with these biologically active CCPs. The present study provides further understanding of the important roles of the hydrophilic and hydrophobic hCCPs (as the MCs) in combination with the non-hCCPs in nervous system intoxication after exposure to harmful cyanobacteria. The patients who developed signs of neurotoxicity in the tragic hepatotoxic poisoning by MCs that occurred in Caruaru in 1996 were exposed to a complex mixture of CCPs. In such cases, even in the absence of the specific OATPs/SLCOs transporters, the non-hCCPs can show similar toxicities to the MCs, in terms of their effects on normal astrocytes, and also at higher concentrations on tumor astrocytes. This cytotoxicity appears to be due to the interactions of the CCPs with the cell membrane and possibly with yet-to-be-defined receptors. We have demonstrated that intermediate filaments and intermediate filament-linking elements are also important targets for the biological activities of the non-hCCPs on astrocytic cells.

## 4. Conclusions

There is a need to protect drinking water sources and recreational water bodies from the health hazards caused by cyanobacteria and their toxins. Outbreaks of human poisoning attributed to toxic cyanobacteria have been reported worldwide and are mostly connected to the impact on human health. Therefore, detailed research on their molecular mechanisms is crucial for an understanding of their systemic toxicity. Our study provides significant insights into the toxicological impact of CCPs on astrocytes in vitro. By examining the effects of non-hepatotoxic PP BL1125 and AnPs -B and -F, as well as hepatotoxic MC-LR, we demonstrated their ability to insert into the lipid monolayer and impair cellular viability. The findings highlight the peptides’ capacity to inhibit Ser/Thr phosphatases, leading to cytoskeleton disruption at both the gene expression level and the organization of cytoskeletal elements, resulting in subsequent communication failures within and between cells.

Therefore, this study emphasizes the possible role of non-hCCPs in systemic intoxication, together with the hydrophilic and hydrophobic CCPs (microcystin family). Even in the absence of the specific OATPs/SLCO transport molecules in these normal and tumor astrocyte cell lines, the non-hCCPs showed similar toxic effects to those of the hCCPs. This cytotoxicity appears to be due to the interactions of the cyanopeptides with the cells and on their membranes and membrane receptors. The lack of cell-to-cell contacts in most of such cultured cell lines also explains the lack of desmoplakin detection. Along with other cases of fatal microcystin liver intoxication, where the loss of cell-to-cell contacts is the consequence of desmoplakin reorganization [26], this case is similar, where the loss of cell-to-cell contacts due to nestin reorganization appears to be the important cause of the CCP neural systemic intoxication. We can deduce that the selected non-hCCP might compensate for their lower Ser/Thr protein phosphatase activity in comparison to MCs by high membrane insertion pressures. However, in addition to the intermediate filaments in correlation to PPs inhibition, the important target of non-hCCPs might also be the tubulin cytoskeleton, either directly or indirectly, due to the high carboxypeptidase inhibition, affecting tubulin post-translational modifications.

## 5. Materials and Methods

### 5.1. Cyclic Cyanopeptides

The hCCP [D-Asp3]-microcystin-RR, and the non-hCCPs PP BL1125, AnP-B and AnP-F were isolated from the field sample of the bloom-forming cyanobacterium Planktothrix rubescens (D.C. ex Gomont) using reverse phase extraction, as described previously [19,54,71]. Microcystin-LR was from Enzo Life Science (Alexis Biochemicals/Enzo Life Sciences, Inc., Farmingdale, NY, USA). Stock solutions at 10 mM were prepared, with each MC dissolved in 100% methanol and each non-hCCP dissolved in 10% methanol, respectively. Solvent (negative) control was employed in each further experiment.

### 5.2. Interactions of CCPs with Lipid Monolayers

Lateral pressure measurements were carried out in a Langmuir trough (MicroTrough-S system; Kibron, Helsinki, Finland) at room temperature (25 °C) under constant stirring. The aqueous sub-phase was 500 µL 10 mM Hepes, 200 mM NaCl, and pH 7.5. First, we determined the concentrations of the CCPs where the air–water interface begins to saturate using PP BL1125 in the absence of lipids. Different concentrations of PP BL1125 (0–30 µM) were injected into the sub-phase, and the change in surface pressure that originated from the interactions of PP BL1125 with the air–water interface was recorded.

To create lipid monolayers, dioleylposphatidylcholine and cholesterol (70:30) were dissolved together in chloroform/methanol (2:1, *v*/*v*) and gently spread over the sub-phase. The desired initial surface pressure was obtained by changing the amounts of the lipid mixture applied to the air–water interface. After solvent evaporation (10 min), each CCP was separately injected through a hole that was connected to the sub-phase, at a final concentration in the Langmuir trough of 20 µM. The changes in surface pressure versus time were recorded until a stable signal was obtained. The critical pressure was defined as the initial pressure at which no more CCPs could be inserted into the monolayer, and this was calculated for PP BL1125 and AnP-B by recording the increases in the surface pressures after injection of the CCPs under test at different initial lipid surface pressures. The increase in the surface pressure (∆Π) was plotted versus the initial pressure (Π_0_), and the critical pressures were determined using linear regression with Prism 5 software (GraphPad, Inc., La Jolla, CA, USA).

### 5.3. Protein Phosphatase Inhibition Assay

The inhibition of Ser/Thr protein phosphatases by the CCPs was measured using the colorimetric inhibition assay described by An and Carmichael [72] and Gkelis et al. [21], with some modifications. Ser/Thr protein phosphatase type-1 (PP1; rabbit recombinant α isoform of PP1 catalytic subunit expressed in *Escherichia coli*; Sigma, St. Louis, MO, USA, Cat. #539493) and Ser/Thr protein phosphatase type-2A (PP2A; from bovine kidney; Sigma, Cat. #P1868) were used in the in vitro protein phosphatase assays, which were carried out in triplicate in 96-well flat-bottomed microplates (Corning Costar) using *p*-nitrophenyl phosphate as substrate (Sigma, St. Louis, MO, USA). Enzymes, PP1 and PP2A, were diluted in reaction buffer #1 (50 mM Tris-HCl, 1.0 g/L bovine serum albumin, 1.0 mM MnCl_2_, 2.0 mM dithiothreitol, pH 7.4), and *p*-nitrophenyl phosphate was prepared in substrate buffer (0.1 M glycine, pH 10.4, 1 mM MgCl_2_, 1 mM ZnCl_2_). The assay was performed by the addition of each CCP (as a potential inhibitor; final concentrations, 0.1, 1, 10, and 100 µM) to the enzyme solution (i.e., PP1 and PP2A; final concentrations, 1 U/well and 0.01 U/well, respectively, where 1 U hydrolyzes 1 nmole of substrate per minute at pH 7.4 at 30 °C). After a few seconds of gentle shaking, the microplate was kept at room temperature for 5 min, followed by the addition of reaction buffer #2 (50 mM Tris-HCl, 0.5 g/L bovine serum albumin, 0.2 mM MnCl_2_, 20 mM MgCl_2_, and pH 8.1) for PP1, or reaction buffer #1 for PP2A. The reactions were started by the addition of *p*-nitrophenyl phosphate (final concentrations, 15 mM for PP1 and 10 mM for PP2A) and were carried out for 120 min at 37 °C. The hydrolysis of *p*-nitrophenyl phosphate to *p*-nitrophenyl was recorded at 405 nm using a microplate reader (Tecan, Austria). The inhibition percentages (*X*) were calculated from the absorbances of the samples after incubation for 30 min, as according to Equation (1):

*X* (%) = (*A_PP_* − *A_S_*)/*A_PP_* × 100, (1)

where *A_PP_* is the absorbance of the control sample (i.e., enzyme only), and *A_S_* is the absorbance of the test sample (i.e., enzyme with potential inhibitor). The half maximal inhibitory concentrations (IC_50_, the concentration of inhibitor, e.g., PP BL1125, AnP-B, AnP-F, and MC-LR) required to produce 50% inhibition of PP1 and PP2A at specific substrate concentration) were calculated using Prism 5 software (GraphPad, Inc., La Jolla, CA, USA) and non-linear regression curve fit, described with dose-response–inhibition equation log(inhibition) vs. response. All measurements were repeated a minimum of three independent times, with five technical replicates per experiment. IC_50_ values are expressed as means ± SD.

### 5.4. Cell Culture

Normal human astrocytes (NHAs; Lonza, Walkersville, MD, USA) were grown and maintained in monolayer cultures in Dulbecco’s modified Eagle’s medium (DMEM; Sigma) supplemented with 10% fetal bovine serum (Euroclone, Pero, MI, Italy), 1% penicillin/streptomycin (Euroclone), 2 mM L-glutamine (Euroclone), and 20 mM Hepes (Invitrogen, Carlsbad, CA, USA). The U87-MG human glioblastoma cells (U87-MG cells; American Type Culture Collection, Manassas, VA, USA) were grown and maintained in monolayer cultures in DMEM supplemented with 10% fetal bovine serum, 1% penicillin/streptomycin, 4 mM L-glutamine, and 1% non-essential amino acids (Sigma).

All the cell lines were plated in cell culture flasks (T-150; Corning Costar, Cambridge, MS, USA) and incubated in a standard CO_2_ incubator at 37 °C and in a 5% CO_2_ atmosphere (Kambič, Semič, Slovenia). Once they had reached 80% confluence, the cells were trypsinized (0.25% trypsin/0.2% EDTA; Invitrogen, Waltham, MA, USA), stained with Trypan blue (Sigma-Aldrich, St. Louis, MO, USA), and counted using a hemocytometer (BRAND, Wertheim, Germany). The use of dimethylsulphoxide was strictly avoided in these cell culture experiments, as it can influence the permeability of cell membranes to CCPs (except for the dissolution of formazan crystals in the cell viability assays).

### 5.5. Metabolic Activity of the Cells

Cell viability was determined using the 3-(4,5-dimethylthiazolyl-2)-2,5-diphenyltetrazolium bromide (MTT) assay, as according to Mosmann [73]. This assay measures the conversion of MTT to its insoluble formazan by the dehydrogenase enzymes of the intact mitochondria of living cells. The cells were plated in 96-well microplates (3000/well for NHAs, U87-MG cells; Nunclon; Nunc, Naperville, IL, USA) and incubated for 16 h at 37 °C for attachment. The cell-growth medium was then replaced by fresh medium containing 0, 0.1, 1, and 10 µM of each CCP and incubated for an additional 24 h. These experiments were performed without using dimethylsulphoxide to best retain the original permeability of the cell membranes. Each experiment included the negative control of cell-growth medium with no additions. Following incubation (CO_2_ incubator), 20 μL 5 mg/mL MTT was added to each well. The plates were incubated for another 3 h. The medium was then removed, and 200 μL dimethylsulphoxide/well was added to dissolve the formazan crystals, which resulted in a dark blue solution. The amount of formazan, which is proportional to the metabolic activity of cells, was quantified spectrophotometrically at 570 nm (reference filter wavelength, 690 nm) using a microplate reader (Synergy MX; BioTek, Winooski, VT, USA). The metabolic activity of the cells was determined by comparing the optical densities of the CCP-treated cells with those of the negative control. Five replicates per concentration and three independent experiments were performed. Student’s *t*-tests were used to analyze the differences between the treated and control cells using Prism 5 software (GraphPad, Inc., La Jolla, CA, USA), where *p* < 0.05 was considered to be statistically significant.

### 5.6. mRNA Expression Analysis

Cells were seeded onto 6-well plates, left to attach for 16 h, and treated with each non-hCCP separately (0.1–10 µM) and the hepatotoxic MC-LR (0.01–1 µM) for 24 h at 37 °C in the CO_2_ incubator. The total RNA from the cells (0.5–1.0 × 10^6^ cells) was isolated using the TRIzol reagent (Invitrogen), and the cDNA was synthesized using 1 μg total RNA with cDNA high-capacity archive kits (Applied Biosystems, Foster City, CA, USA), according to the manufacturer’s protocol.

The expression levels of *TUBA1A*, *ACTB*, *NES*, and *GFAP* were quantified using quantitative real-time PCR (ABI 7900 HT Sequence Detection System; Applied Biosystems). TaqMan Universal PCR Master Mix and the following TaqMan Gene Expression Assays were used: *TUBA1A*: tubulin, alpha 1a, Hs00362387_m1; *ACTB*: actin, beta, Hs99999903_m1; *NES*, nestin, Hs00707120_s1; and *GFAP*, glial fibrillary acidic protein, and Hs00157674_m1 (all from Applied Biosystems). Amplification of a glyceraldehyde 3-phosphate dehydrogenase probe *GAPDH* (Human Endogenous Controls, Cat. # 4310884E; Applied Biosystems) was performed as an internal control. The conditions for the quantitative real-time PCR were as follows: 50 °C for 2 min, 95 °C for 10 min, 40 cycles of 95 °C for 15 s, and 60 °C for 1 min.

The data obtained from the TaqMan Gene Expression Assays were analyzed using the ΔΔCt algorithm. Two replicates per concentration and three independent experiments were performed. The statistical significance between the controls and the treated groups was determined using two-tailed Student’s *t*-tests, where *p* < 0.05 was statistically significant. In addition, the relative expression was normalized to the housekeeping gene *GAPDH* (relative quantification), with at least three sample concentrations used for each gene in both untreated cell lines.

To confirm the expression of the OATP/SLCO transporters in the NHAs and U87-MG cells, gene expression analyses for *SLCO1A2* (solute carrier organic anion transporter family member 1A2, Hs00366488_m1), *SLCO1B1* (solute carrier organic anion transporter family member 1B1, Hs00272374_m1), and *SLCO1B3* (solute carrier organic anion transporter family member 1B3, Hs00251986_m1) (all from Applied Biosystems) were carried out using quantitative real-time PCR, as described above.

### 5.7. Cytoskeleton Immunostaining

To determine the influence of the CCPs on actin microfilament and microtubule organization, the cells were labeled with rhodamine-conjugated phalloidin (Molecular Probes, Cat. # R415), and mouse monoclonal anti-bovine α-tubulin antibodies (Molecular Probes Cat. # A11126) and secondary Alexa-488 goat anti-mouse IgG (Molecular Probes Cat. # A-11017), respectively. The cells (3000/well) were plated into 16-well plates (Culture Well Chambered Coverglass for Cell Culture; Molecular Probes Inc., Eugene, OR, USA) in 100 μL DMEM. After incubation in a CO_2_ incubator at 37 °C and in a 5% CO_2_ atmosphere for 16 h, the cells were treated with the selected non-hCCPs (PP BL1125, AnP-B, and AnP-F) using the [D-Asp^3^]- as the positive control [54,74] with the analysis of two concentrations of each CCP separately (10 and 20 µM).

For the intermediate filaments, visualization at 1 µM MC-LR was used as the positive control. The cells were stained with rabbit monoclonal anti-GFAP (Merck Millipore, Bedford, MA, USA, Cat. # AB5804) and secondary Alexa-488 goat anti-rabbit IgG (Molecular Probes, Cat. # A-11034) antibodies and with mouse monoclonal anti-nestin (Merck Millipore, Cat. # MAB5326) and secondary Alexa-488 goat anti-mouse IgG (Molecular Probes, Cat. # A-11017) antibodies.

The desmosomes in both cells were labeled using immunocytochemical detection of desmoplakin I+II, two main components of desmosomal plaques, using anti-desmoplakin I+II antibodies (Abcam, Cambridge, MA, USA, Cat. # ab16434). HepG2 human hepatoma cells (European Collection of Cell Cultures, Salisbury, UK) were used as the positive control for desmosomes. Each staining experiment was carried out with at least two parallel measurements for each concentration of CCP tested.

The cells in all the experiments were exposed to the CCPs for an additional 24 h in the CO_2_ incubator at 37 °C in a 5% CO_2_ atmosphere before they were fixed.

Fixing and staining. The cells were washed in phosphate-buffered saline (PBS) and fixed in 3.7% paraformaldehyde in either PBS (37 °C, 30 min; for microfilaments, microtubules, nestin) or in ice-cold methanol (4 °C, 10 min; for GFAP, desmoplakin I+II). Then the cells were permeabilized in PBS containing 0.1% Triton X-100 (*v*/*v*; Merck, Germany) for 20 min and blocked in 4% bovine serum albumin (Sigma) at room temperature for 10 min. For desmoplakin I+II visualization, the cells were blocked in 10% fetal bovine serum (Sigma) for 60 min without cell permeabilization. Microfilaments were stained with rhodamine-conjugated phalloidin (1:40) for 30 min at room temperature. Microtubules were visualized by immunostaining using an anti-α-tubulin antibody overnight at 4 °C (1:200 in PBS containing Ca^2+^, 0.1% [*v*/*v*] Tween-20) and the secondary antibodies labeled with Alexa-488 goat anti-mouse IgG (1:1000; 30 min at room temperature). Intermediate filaments were stained using anti-nestin (1:500; overnight at 4 °C) and anti-GFAP (1:500, overnight at 4 °C) antibodies. The anti-nestin-labeled cells were visualized using secondary antibodies labeled with Alexa-488 goat anti-mouse IgG and the anti-GFAP-labeled cells with Alexa-488 anti-rabbit IgG (1:1000 dilution, room temperature, 30 min). Desmoplakin I+II were stained with a mouse anti-desmoplakin I+II antibody (1:500 dilution; room temperature, 60 min) and visualized with Alexa-488 goat anti-mouse IgG (1:1000 dilution; room temperature, 30 min). The cell nuclei were stained with To-Pro-3 (Molecular Probes; 1:1000 dilution; room temperature, 30 min). After each step, the samples were washed three times with PBS. Finally, all the cell preparations were immersed in an antifade reagent (Prolong Gold; Molecular Probes) to prevent photobleaching.

Microscopy. The cells were examined using a multispectral laser scanning confocal microscope (Leica TCS NT; Leica Lasertechnik, Wetzlar, Germany), with an argon ion laser beam at 488 nm, and a helium–neon laser at 543 nm and 633 nm. The fluorescent light was channeled to the photomultiplier tube through a dichroic prism (488/543/633), and a 1 μm pinhole was used to create the confocal image. The cell preparations were also examined under light microscopy (Eclipse TE 3000; Nikon, Japan), and the images were recorded under phase contrast and epifluorescence (i.e., B-2A and G-2A filters, respectively).

## Figures and Tables

**Table 1:** Ser/Thr protein phosphatase PP1 and PP2A inhibition by the three non-hCCPs (PP BL1125, AnP-B, and AnP-F) and the hCCP MC-LR, expressed as IC_50_. Phosphatases inhibition was measured using the colorimetric inhibition assay with *p*-nitrophenyl phosphate as substrate and are expressed as IC_50_. The IC_50_ values were calculated using Prism 5 software (GraphPad, Inc., La Jolla, CA, USA) and non-linear regression curve fit, described with dose-response—inhibition equation log(inhibition) vs. response. All measurements were repeated a minimum of three independent times, and IC_50_ values are expressed as means ± SD.

CCP	IC_50_ (µM)	
	PP1	PP2A
PP BL1125	131.5 ± 87.5	18.2 ± 15.7
AnP-B	9.5 ± 0.9	12.3 ± 6.1
AnP-F	28.2 ± 3.4	1.0 ± 0.2
MC-LR	0.0050 ± 0.0016	0.000048 ± 0.00001

**Table 2:** Changes in gene expression of TUBA1A, ACTB, NES, and GFAP in NHA (**A**) and U-87MG cells (**B**) after exposure to CCPs (0.1–10 µM) for 24 h.

(A)					
Gene	CCP (µM)	PP BL1125	AnP-B	AnP-F	MC-LR
*TUBA1A*	0	1.005 ± 0.013	1.005 ± 0.013	1.005 ± 0.013	1.004 ± 0.097
	0.1	0.940 ± 0.162	1.082 ± 0.219	0.941 ± 0.186	0.957 ± 0.046
	1	0.824 ± 0.128 **	0.807 ± 0.100 ***	1.144 ± 0.291	0.941 ± 0.025
	10	1.000 ± 0.141	0.895 ± 0.119 *	0.826 ± 0.036 ***	/
*ACTB*	0	1.010 ± 0.022	1.010 ± 0.022	1.010 ± 0.022	1.016 ± 0.204
	0.1	0.949 ± 0.140	1.175 ± 0.386	0.824 ± 0.227	1.071 ± 0.437
	1	0.988 ± 0.147	0.935 ± 0.157	1.121 ± 0.167	1.866 ± 0.844
	10	1.162 ± 0.251	0.934 ± 0.166	0.890 ± 0.184	/
*NES*	0	1.001 ± 0.002	1.001 ± 0.002	1.001 ± 0.002	1.032 ± 0.299
	0.1	1.262 ± 0.077 ***	1.361 ± 0.291 *	1.345 ± 0.205 **	0.917 ± 0.067
	1	1.203 ± 0.196 *	1.221 ± 0.338	1.449 ± 0.171 ***	1.266 ± 0.250
	10	1.370 ± 0.208 **	1.388 ± 0.313 *	1.381 ± 0.173 ***	/
*GFAP*	0	1.011 ± 0.019	1.011 ± 0.019	1.011 ± 0.019	1.086 ± 0.503
	0.1	1.051 ± 0.351	1.053 ± 0.124	1.326 ± 0.507	1.395 ± 0.312
	1	0.947 ± 0.101	1.434 ± 0.180 **	0.846 ± 0.211	1.453 ± 0.279
	10	1.119 ± 0.240	0.918 ± 0.204	1.167 ± 0.250	/
**(B)**					
**Gene**	**CCP (µM)**	**PP BL1125**	**AnP-B**	**AnP-F**	**MC-LR**
*TUBA1A*	0	1.001 ± 0.002	1.001 ± 0.002	1.001 ± 0.002	1.001 ± 0.001
	0.1	1.124 ± 0.050 *	1.131 ± 0.031 **	1.016 ± 0.098	1.031 ± 0.064
	1	1.094 ± 0.172	1.098 ± 0.192	1.023 ± 0.160	0.983 ± 0.099
	10	1.171 ± 0.049 **	1.429 ± 0.068 ***	1.354 ± 0.126 **	/
*ACTB*	0	1.003 ± 0.005	1.003 ± 0.005	1.003 ± 0.005	1.002 ± 0.002
	0.1	1.015 ± 0.057	0.998 ± 0.070	0.935 ± 0.118	1.466 ± 0.286 **
	1	1.051 ± 0.185	1.039 ± 0.070	1.084 ± 0.138	1.796 ± 0.277 ***
	10	1.147 ± 0.105	1.122 ± 0.033 **	1.046 ± 0.130	/
*NES*	0	1.000 ± 0.001	1.000 ± 0.001	1.000 ± 0.001	1.001 ± 0.001
	0.1	1.003 ± 0.164	0.948 ± 0.143	1.040 ± 0.105	1.030 ± 0.105
	1	1.069 ± 0.105	0.992 ± 0.085	1.081 ± 0.056	0.877 ± 0.148
	10	1.240 ± 0.041 **	0.931 ± 0.054	0.886 ± 0.037 **	/
*GFAP*	0	1.010 ± 0.015	1.010 ± 0.015	1.010 ± 0.015	1.005 ± 0.005
	0.1	1.107 ± 0.214	1.088 ± 0.390	1.002 ± 0.087	0.986 ± 0.120
	1	1.283 ± 0.185	1,109 ± 0.215	1.096 ± 0.211	0.909 ± 0.052 **
	10	1.016 ± 0.046	1.175 ± 0.148	1.191 ± 0.435	/

* A significant difference between CCP-treated groups and the non-treated control (Student’s test: *p* < 0.05); ** a significant difference between CCP-treated groups and the non-treated control (Student’s test: *p* < 0.01); *** a significant difference between CCP-treated groups and the non-treated control (Student’s test: *p* < 0.001);/concentration not tested.

## Data Availability

The raw data supporting the conclusions of this article will be made available by the authors on request.

## Supplementary Materials

The following supporting information can be downloaded at https://www.mdpi.com/article/10.3390/toxins16090374/s1: Figure S1: Effects of CCPs on relative viability of NHAs (**A**) and U87-MG cells (**B**). Cell viability was determined using the MTT assay after exposure to the CCPs as indicated, at the concentrations indicated, for 24 h. * *p* < 0.05 vs. relative control (Student’s *t*-tests).; Figure S2: Relative expression of cytoskeletal genes in NHAs (**A**) and the U87-MG cells (**B**). Expression levels of the indicated genes were quantified using quantitative real-time PCR. Data are expressed as relative expression normalized to the housekeeping gene GAPDH. *nd*, not determined (gene expression too low).

## References

[1] Cheung M.Y., Liang S., Lee J. (2013). Toxin-Producing Cyanobacteria in Freshwater: A Review of the Problems, Impact on Drinking Water Safety, and Efforts for Protecting Public Health. J. Microbiol..

[2] Paerl H.W. (2014). Mitigating Harmful Cyanobacterial Blooms in a Human- and Climatically-Impacted World. Life.

[3] Chorus I., Fastner J., Welker M. (2021). Cyanobacteria and Cyanotoxins in a Changing Environment: Concepts, Controversies, Challenges. Water.

[4] Moreira C., Vasconcelos V., Antunes A. (2022). Cyanobacterial Blooms: Current Knowledge and New Perspectives. Earth.

[5] Sauvé S., Desrosiers M. (2014). A Review of What Is an Emerging Contaminant. Chem. Cent. J..

[6] Pouria S., De Andrade A., Barbosa J., Cavalcanti R.L., Barreto V.T.S., Ward C.J., Preiser W., Poon G.K., Neild G.H., Codd G.A. (1998). Fatal Microcystin Intoxication in Haemodialysis Unit in Caruaru, Brazil. Lancet.

[7] Yuan M., Carmichael W.W., Hilborn E.D. (2006). Microcystin Analysis in Human Sera and Liver from Human Fatalities in Caruaru, Brazil 1996. Toxicon.

[8] Welker M., Brunke M., Preussel K., Lippert I., Von Döhren H. (2004). Diversity and Distribution of Microcystis (Cyanobacteria) Oligopeptide Chemotypes from Natural Communities Studied by Single-Colony Mass Spectrometry. Microbiology.

[9] Ding W.-X., Shen H.-M., Zhu H.-G., Lee B.-L., Ong C.-N. (1999). Genotoxicity of Microcystic Cyanobacteria Extract of a Water Source in China. Mutat. Res./Genet. Toxicol. Environ. Mutagen..

[10] Honkanen R.E., Zwiller J., Moore R.E., Daily S.L., Khatra B.S., Dukelow M., Boynton A.L. (1990). Characterization of Microcystin-LR, a Potent Inhibitor of Type 1 and Type 2A Protein Phosphatases. J. Biol. Chem..

[11] Pereira S.R., Vasconcelos V.M., Antunes A. (2011). The Phosphoprotein Phosphatase Family of Ser/Thr Phosphatases as Principal Targets of Naturally Occurring Toxins. Crit. Rev. Toxicol..

[12] Fischer W.J., Altheimer S., Cattori V., Meier P.J., Dietrich D.R., Hagenbuch B. (2005). Organic Anion Transporting Polypeptides Expressed in Liver and Brain Mediate Uptake of Microcystin. Toxicol. Appl. Pharmacol..

[13] Toivola D.M., Eriksson J.E. (1999). Toxins Affecting Cell Signalling and Alteration of Cytoskeletal Structure. Toxicol. Vitr..

[14] Fischer A., Hoeger S.J., Stemmer K., Feurstein D.J., Knobeloch D., Nussler A., Dietrich D.R. (2010). The Role of Organic Anion Transporting Polypeptides (OATPs/SLCOs) in the Toxicity of Different Microcystin Congeners in Vitro: A Comparison of Primary Human Hepatocytes and OATP-Transfected HEK293 Cells. Toxicol. Appl. Pharmacol..

[15] Suchy F.J. (1993). Hepatocellular Transport of Bile Acids. Semin. Liver Dis..

[16] Gehringer M.M. (2004). Microcystin-LR and Okadaic Acid-induced Cellular Effects: A Dualistic Response. FEBS Lett..

[17] Batista T., De Sousa G., Suput J.S., Rahmani R., Šuput D. (2003). Microcystin-LR Causes the Collapse of Actin Filaments in Primary Human Hepatocytes. Aquat. Toxicol..

[18] Eleršek T., Bláha L., Mazur-Marzec H., Schmidt W., Carmeli S. (2016). Other Cyanobacterial Bioactive Substances. Handbook of Cyanobacterial Monitoring and Cyanotoxin Analysis.

[19] Grach-Pogrebinsky O., Sedmak B., Carmeli S. (2003). Protease Inhibitors from a Slovenian Lake Bled Toxic Waterbloom of the Cyanobacterium *Planktothrix rubescens*. Tetrahedron.

[20] Harada K.-I., Fujii K., Shimada T., Suzuki M., Sano H., Adachi K., Carmichael W.W. (1995). Two Cyclic Peptides, Anabaenopeptins, a Third Group of Bioactive Compounds from the cyanobacterium Anabaena Flos-Aquae NRC 525-17. Tetrahedron Lett..

[21] Gkelis S., Lanaras T., Sivonen K. (2006). The Presence of Microcystins and Other Cyanobacterial Bioactive Peptides in Aquatic Fauna Collected from Greek Freshwaters. Aquat. Toxicol..

[22] Bubik A., Sedmak B., Novinec M., Lenarčič B., Lah T.T. (2008). Cytotoxic and Peptidase Inhibitory Activities of Selected Non-Hepatotoxic Cyclic Peptides from Cyanobacteria. Biol. Chem..

[23] Spoof L., Błaszczyk A., Meriluoto J., Cegłowska M., Mazur-Marzec H. (2015). Structures and Activity of New Anabaenopeptins Produced by Baltic Sea Cyanobacteria. Mar. Drugs.

[24] Schreuder H., Liesum A., Lönze P., Stump H., Hoffmann H., Schiell M., Kurz M., Toti L., Bauer A., Kallus C. (2016). Isolation, Co-Crystallization and Structure-Based Characterization of Anabaenopeptins as Highly Potent Inhibitors of Activated Thrombin Activatable Fibrinolysis Inhibitor (TAFIa). Sci. Rep..

[25] Chlipala G.E., Mo S., Orjala J. (2011). Chemodiversity in Freshwater and Terrestrial Cyanobacteria—A Source for Drug Discovery. Curr. Drug Targets.

[26] Toivola D.M., Goldman R.D., Garrod D.R., Eriksson J.E. (1997). Protein Phosphatases Maintain the Organization and Structural Interactions of Hepatic Keratin Intermediate Filaments. J. Cell Sci..

[27] Aillaud C., Bosc C., Peris L., Bosson A., Heemeryck P., Van Dijk J., Le Friec J., Boulan B., Vossier F., Sanman L.E. (2017). Vasohibins/SVBP Are Tubulin Carboxypeptidases (TCPs) That Regulate Neuron Differentiation. Science.

[28] Barlič A., Gutiérrez-Aguirre I., Caaveiro J.M.M., Cruz A., Ruiz-Argüello M.-B., Pérez-Gil J., González-Mañas J.M. (2004). Lipid Phase Coexistence Favors Membrane Insertion of Equinatoxin-II, a Pore-Forming Toxin from Actinia Equina. J. Biol. Chem..

[29] Eiríksdóttir E., Konate K., Langel Ü., Divita G., Deshayes S. (2010). Secondary Structure of Cell-Penetrating Peptides Controls Membrane Interaction and Insertion. Biochim. Et Biophys. Acta (BBA) Biomembr..

[30] Yoshizawa S., Matsushima R., Watanabe M.F., Harada K.-I., Ichihara A., Carmichael W.W., Fujiki H. (1990). Inhibition of Protein Phosphatases by Microcystis and Nodularin Associated with Hepatotoxicity. J. Cancer Res. Clin. Oncol..

[31] Xu S., Yi X., Liu W., Zhang C., Massey I.Y., Yang F., Tian L. (2020). A Review of Nephrotoxicity of Microcystins. Toxins.

[32] Yi X., Xu S., Huang F., Wen C., Zheng S., Feng H., Guo J., Chen J., Feng X., Yang F. (2019). Effects of Chronic Exposure to Microcystin-LR on Kidney in Mice. Int. J. Environ. Res. Public Health.

[33] Lin H., Liu W., Zeng H., Pu C., Zhang R., Qiu Z., Chen J.-A., Wang L., Tan Y., Zheng C. (2016). Determination of Environmental Exposure to Microcystin and Aflatoxin as a Risk for Renal Function Based on 5493 Rural People in Southwest China. Environ. Sci. Technol..

[34] Li X., Xu L., Zhou W., Zhao Q., Wang Y. (2016). Chronic Exposure to Microcystin-LR Affected Mitochondrial DNA Maintenance and Caused Pathological Changes of Lung Tissue in Mice. Environ. Pollut..

[35] Soares R.M., Cagido V.R., Ferraro R.B., Meyer-Fernandes J.R., Rocco P.R.M., Zin W.A., Azevedo S.M.F.O. (2007). Effects of Microcystin-LR on Mouse Lungs. Toxicon.

[36] Wu J.-X., Huang H., Yang L., Zhang X.-F., Zhang S.-S., Liu H.-H., Wang Y.-Q., Yuan L., Cheng X.-M., Zhuang D.-G. (2018). Gastrointestinal Toxicity Induced by Microcystins. World J. Clin. Cases.

[37] Cao L., Huang F., Massey I.Y., Wen C., Zheng S., Xu S., Yang F. (2019). Effects of Microcystin-LR on the Microstructure and Inflammation-Related Factors of Jejunum in Mice. Toxins.

[38] Cao L., Massey I.Y., Feng H., Yang F. (2019). A Review of Cardiovascular Toxicity of Microcystins. Toxins.

[39] Xiong Q., Xie P., Li H., Hao L., Li G., Qiu T., Liu Y. (2010). Acute Effects of Microcystins Exposure on the Transcription of Antioxidant Enzyme Genes in Three Organs (Liver, Kidney, and Testis) of Male Wistar Rats. J. Biochem. Mol. Toxicol..

[40] Milutinović A., Živin M., Zorc-Pleskovič R., Sedmak B., Šuput D. (2003). Nephrotoxic Effects of Chronic Administration of Microcystins -LR and -YR. Toxicon.

[41] Hinojosa M.G., Gutiérrez-Praena D., Prieto A.I., Guzmán-Guillén R., Jos A., Cameán A.M. (2019). Neurotoxicity Induced by Microcystins and Cylindrospermopsin: A Review. Sci. Total Environ..

[42] Hu Y., Chen J., Fan H., Xie P., He J. (2016). A Review of Neurotoxicity of Microcystins. Environ. Sci. Pollut. Res..

[43] Yan M., Jin H., Pan C., Hang H., Li D., Han X. (2022). Movement Disorder and Neurotoxicity Induced by Chronic Exposure to Microcystin-LR in Mice. Mol. Neurobiol..

[44] Runnegar M.T., Kong S., Berndt N. (1993). Protein Phosphatase Inhibition and in Vivo Hepatotoxicity of Microcystins. Am. J. Physiol. Gastrointest. Liver Physiol..

[45] Yang F., Wen C., Zheng S., Yang S., Chen J., Feng X. (2018). Involvement of MAPK/ERK1/2 Pathway in Microcystin-Induced Microfilament Reorganization in HL7702 Hepatocytes. J. Toxicol. Environ. Health Part A.

[46] Shi L., Du X., Liu H., Chen X., Ma Y., Wang R., Tian Z., Zhang S., Guo H., Zhang H. (2021). Update on the Adverse Effects of Microcystins on the Liver. Environ. Res..

[47] Li T., Fan X., Cai M., Jiang Y., Wang Y., He P., Ni J., Mo A., Peng C., Liu J. (2023). Advances in Investigating Microcystin-Induced Liver Toxicity and Underlying Mechanisms. Sci. Total Environ..

[48] Bulc Rozman K., Jurič D.M., Šuput D. (2017). Selective Cytotoxicity of Microcystins LR, LW and LF in Rat Astrocytes. Toxicol. Lett..

[49] Filipič M., Žegura B., Sedmak B., Horvat-Žnidaršič I., Milutinovič A., Šuput D. (2007). Subchronic Exposure of Rats to Sublethal Dose of Microcystin-YR Induces DNA Damage in Multiple Organs. Radiol. Oncol..

[50] Li X.-B., Zhang X., Ju J., Li Y., Yin L., Pu Y. (2014). Alterations in Neurobehaviors and Inflammation in Hippocampus of Rats Induced by Oral Administration of Microcystin-LR. Environ. Sci. Pollut. Res..

[51] Wickstrom M.L., Khan S.A., Haschek W.M., Wyman J.F., Eriksson J.E., Schaeffer D.J., Beasley V.R. (1995). Alterations in Microtubules, Intermediate Filaments, and Microfilaments Induced by Microcystin-LR in Cultured Cells. Toxicol. Pathol..

[52] Vesterkvist P.S.M., Meriluoto J.A.O. (2003). Interaction between Microcystins of Different Hydrophobicities and Lipid Monolayers. Toxicon.

[53] Vesterkvist P.S.M., Misiorek J.O., Spoof L.E.M., Toivola D.M., Meriluoto J.A.O. (2012). Comparative Cellular Toxicity of Hydrophilic and Hydrophobic Microcystins on Caco-2 Cells. Toxins.

[54] Grach-Pogrebinsky O., Sedmak B., Carmeli S. (2004). *Seco*[d-Asp^3^]microcystin-RR and [d-Asp^3^,d-Glu(OMe)^6^]microcystin-RR, Two New Microcystins from a Toxic Water Bloom of the Cyanobacterium *Planktothrix rubescens*. J. Nat. Prod..

[55] Eriksson J.E., Grönberg L., Nygård S., Slotte J.P., Meriluoto J.A.O. (1990). Hepatocellular Uptake of 3H-Dihydromicrocystin-LR, a Cyclic Peptide Toxin. Biochim. Et Biophys. Acta (BBA) Biomembr..

[56] Máthé C., Beyer D., M-Hamvas M., Vasas G. (2016). The Effects of Microcystins (Cyanobacterial Heptapeptides) on the Eukaryotic Cytoskeletal System. Mini Rev. Med. Chem..

[57] Zhou M., Tu W.-W., Xu J. (2015). Mechanisms of Microcystin-LR-Induced Cytoskeletal Disruption in Animal Cells. Toxicon.

[58] Toivola D.M., Eriksson J.E., Brautigan D.L. (1994). Identification of Protein Phosphatase 2A as the Primary Target for microcystin-LR in Rat Liver Homogenates. FEBS Lett..

[59] Contín M.A., Purro S.A., Bisig C.G., Barra H.S., Arce C.A. (2003). Inhibitors of Protein Phosphatase 1 and 2A Decrease the Level of Tubulin Carboxypeptidase Activity Associated with Microtubules. Eur. J. Biochem..

[60] Panda D., DeLuca K., Williams D., Jordan M.A., Wilson L. (1998). Antiproliferative Mechanism of Action of Cryptophycin-52: Kinetic Stabilization of Microtubule Dynamics by High-Affinity Binding to Microtubule Ends. Proc. Natl. Acad. Sci. USA.

[61] Pekny M., Lane E.B. (2007). Intermediate Filaments and Stress. Exp. Cell Res..

[62] Pekny M., Nilsson M. (2005). Astrocyte Activation and Reactive Gliosis. Glia.

[63] Toivola D.M., Omary M.B., Ku N., Peltola O., Baribault H., Eriksson J.E. (1998). Protein Phosphatase Inhibition in Normal and Keratin 8/18 Assembly-Incompetent Mouse Strains Supports a Functional Role of Keratin Intermediate Filaments in Preserving Hepatocyte Integrity. Hepatology.

[64] Herrmann H., Aebi U. (2000). Intermediate Filaments and Their Associates: Multi-Talented Structural Elements Specifying Cytoarchitecture and Cytodynamics. Curr. Opin. Cell Biol..

[65] Toivola D.M., Tao G.-Z., Habtezion A., Liao J., Omary M.B. (2005). Cellular Integrity plus: Organelle-Related and Protein-Targeting Functions of Intermediate Filaments. Trends Cell Biol..

[66] Eriksson J.E., Paatero G.I.L., Meriluoto J.A.O., Codd G.A., Kass G.E.N., Nicotera P., Orrenius S. (1989). Rapid Microfilament Reorganization Induced in Isolated Rat Hepatocytes by Microcystin-LR, a Cyclic Peptide Toxin. Exp. Cell Res..

[67] Hooser S.B., Beasley V.R., Waite L.L., Kuhlenschmidt M.S., Carmichael W.W., Haschek W.M. (1991). Actin Filament Alterations in Rat Hepatocytes Induced In Vivo and In Vitro by Microcystin-LR, a Hepatotoxin from the Blue-Green Alga, *Microcystis aeruginosa*. Vet. Pathol..

[68] Runnegar M.T.C., Falconer I.R. (1986). Effect of Toxin from the Cyanobacterium Microcystis Aeruginosa on Ultrastructural Morphology and Actin Polymerization in Isolated Hepatocytes. Toxicon.

[69] Jordan M.A., Wilson L. (2004). Microtubules as a Target for Anticancer Drugs. Nat. Rev. Cancer.

[70] Volterra A., Meldolesi J. (2005). Astrocytes, from Brain Glue to Communication Elements: The Revolution Continues. Nat. Rev. Neurosci..

[71] Sedmak B., Eleršek T., Grach-Pogrebinsky O., Carmeli S., Sever N., Lah T. (2008). Ecotoxicologically Relevant Cyclic Peptides from Cyanobacterial Bloom (Planktothrix Rubescens)—A Threat to Human and Environmental Health. Radiol. Oncol..

[72] An J., Carmichael W.W. (1994). Use of a Colorimetric Protein Phosphatase Inhibition Assay and Enzyme Linked Immunosorbent Assay for the Study of Microcystins and Nodularins. Toxicon.

[73] Mosmann T. (1983). Rapid Colorimetric Assay for Cellular Growth and Survival: Application to Proliferation and Cytotoxicity Assays. J. Immunol. Methods.

[74] Harada K.-I., Matsuura K., Suzuki M., Watanabe M.F., Oishi S., Dahlem A.M., Beasley V.R., Carmichael W.W. (1990). Isolation and Characterization of the Minor Components Associated with Microcystins LR and RR in the Cyanobacterium (Blue-Green Algae). Toxicon.

