# Supplementary material for: Cyanobacterial Cyclic Peptides Can Disrupt Cytoskeleton Organization in Human Astrocytes—A Contribution to the Understanding of the Systemic Toxicity of Cyanotoxins
Source: Toxins (Basel). 2024 Aug 23;16(9):374. doi: 10.3390/toxins16090374 (PMC11436104; doi:10.3390/toxins16090374)
Supplement: Supplementary file 1 [file toxins-16-00374-s001.zip › toxins-3153560-supplementary.pdf]

toxins

Supplementary Materials: Cyanobacterial Cyclic Peptides Can Disrupt Cytoskeleton Organization in Human Astrocytes—A Contribution to the Understanding of the Systemic Toxicity of Cyanotoxins

Anja Bubik, Robert Frangež, Monika C. Žužek, Ion Gutiérrez-Aguirre, Tamara T. Lah and Bojan Sedmak

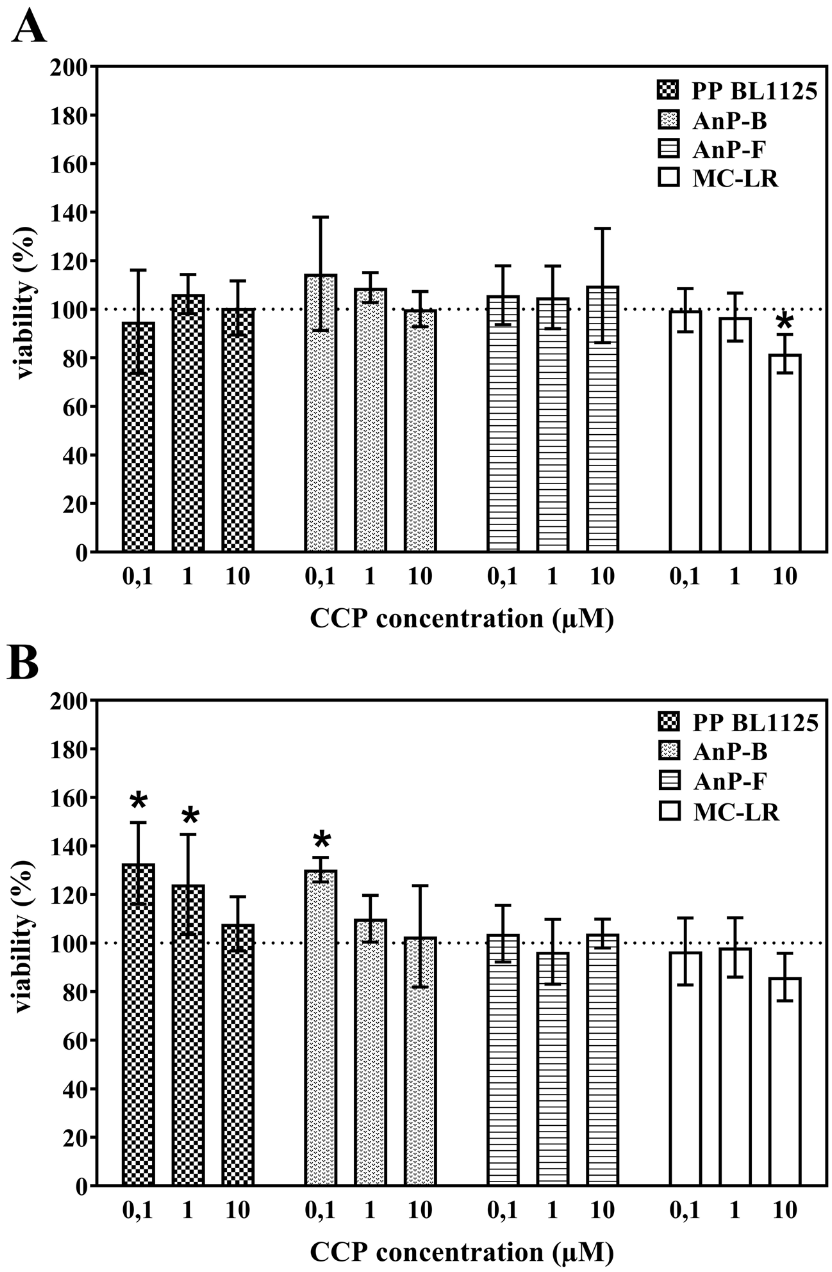

Figure S1. Effects of CCPs on relative viability of NHAs (a) and U87-MG cells (b). Cell viability was determined using the MTT assay after exposure to the CCPs as indicated, at the concentrations indicated, for 24 h. * $p < 0.05$ vs. relative control (Student's *t*-tests).

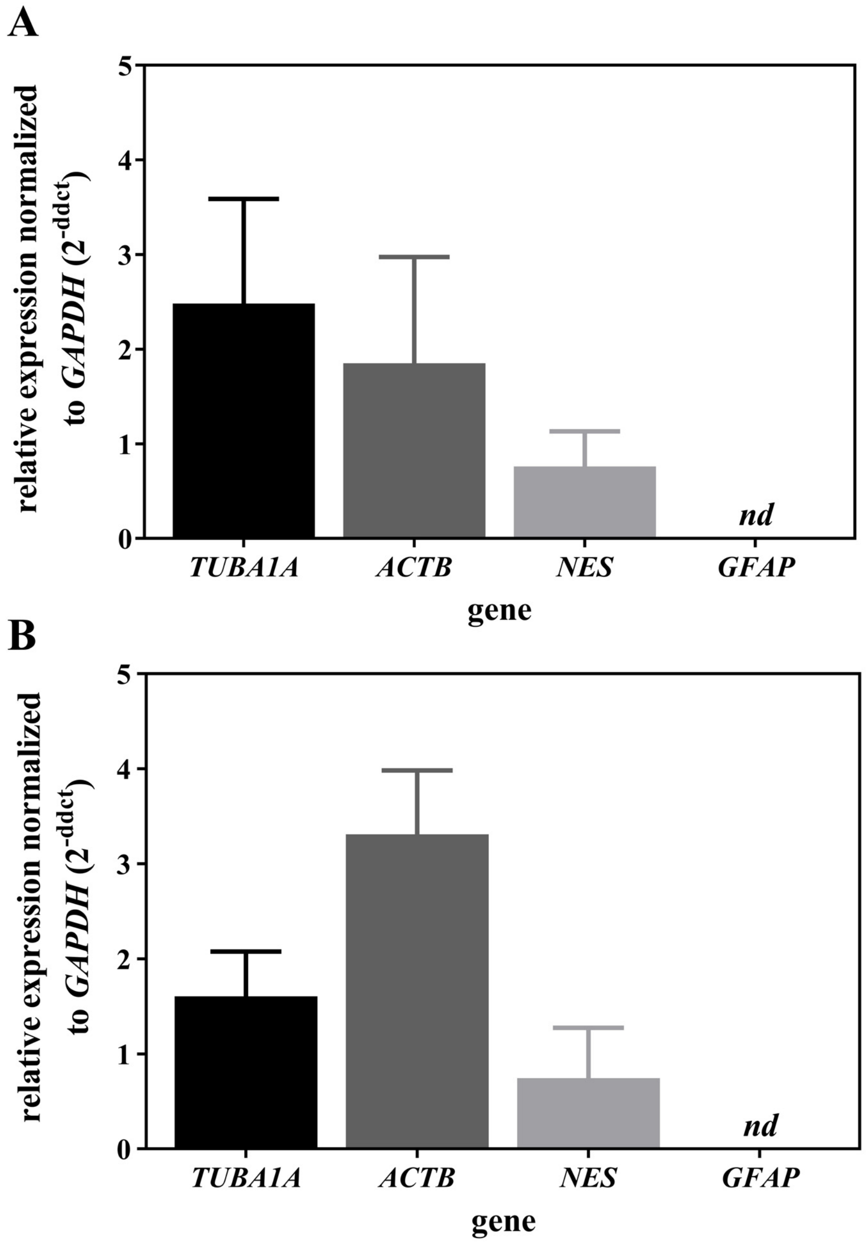

Figure S2. Relative expression of cytoskeletal genes in NHAs (a) and the U87-MG cells (b). Expression levels of the indicated genes were quantified using quantitative real-time PCR. Data are expressed as relative expression normalized to the housekeeping gene *GAPDH*. *nd*, not determined (gene expression too low).
